# The complete mitochondrial genome of *Cimbex luteus* (Hymenoptera: Cimbicidae) and phylogenetic analysis

**DOI:** 10.1080/23802359.2021.1907800

**Published:** 2021-06-21

**Authors:** Yuchen Yan, Ke Li, Siyi Liu, Gengyun Niu, Meicai Wei

**Affiliations:** a Key Laboratory of Insect Evolution and Pest Management for Higher Education in Hunan Province, Central South University of Forestry and Technology, Changsha, PR China; b Jiangxi Normal University, Nanchang, PR China

**Keywords:** Mitogenome, gene rearrangement, phylogeny, Cimbicidae, Cimbex

## Abstract

The complete mitochondrial genome of *Cimbex luteus* was sequenced with 15,127 bp in length. The mitogenome includes 13 protein-coding genes, 22 transfer RNAs, two ribosomal RNAs genes, and an AT-rich region. The nucleotide compositions of *C. luteus* (43.7% A, 38.0% T, 7.4% G, and 10.9% C) were biased toward A and T. Based on Bayesian inference (BI) and Maximum Likelihood (ML) analyses, *C. luteus* was identified as one of the basal lineages of family Cimbicidae.

*Cimbex luteus* is a common pest of Salicaceae Mirb, widely distributed in Palaearctic (Taeger et al. 2010; Hara and Shinohara 2000). Here, we describe the complete mitochondrial genome of *C. luteus* to obtain basic genetic information and advance our understanding of the phylogenetic status of *Cimbex* amongst the family Cimbicidae.

Samples of *C. luteus* were collected in Nanguan District, Changchun City, Jilin Province, China (43.49°N, 125.22°E) in 2017. The specimens (CSCS-Hym-MC0035) were deposited in the Insect Collection of Central South University of Forestry and Technology, Changsha, Hunan, China (CSCS). Total genomic DNA was extracted from single-leg or muscles of adult specimens stored in ethanol at −20 °C using the DNeasy Blood & Tissue Kit (Qiagen, Hilden, Germany). The second generation sequencing and bioinformatic analyses were performed by Shanghai Majorbio Bio-pharm Technology Co., Ltd, yielding a total of 33,247,000 raw reads (SRR12816458). DNA sequences were assembled using MitoZ (Meng et al. 2019*)*, and Geneious Prime version 2019.2.1 (https://www.geneious.com). The genes of *C. luteus* were predicted using online tool MITOS (http://mitos.bioinf.uni-leipzig.de/index.py) (Bernt et al. 2013) with invertebrate mitochondrial code as the genetic code. All genes were further validated using Geneious Prime.

A complete mitogenome of 15,127 bp was obtained and deposited in GenBank with accession number MW136447. The mitochondrial genome of *C. luteus* contains 13 protein-coding genes, 22 transfer RNAs, two ribosomal RNAs genes, and an AT-rich control region. Gene order was conserved and identical to most other previously sequenced Cimbicidae (Yan et al. 2019). The overall mitochondrial base composition of this genome was 43.7% A, 38.0% T, 7.4% G, and 10.9% C, with a A + T content of 81.7%. The size of tRNA varies from 65 (*trnI* and *trnN*) to 71 bp (*trnK*). The *rrnL* gene of *C. luteus* was 1,340 bp in length with A + T content of 84.8%, while *rrnS* was 792 bp in length with A + T content of 84.4% .

Phylogenetic tree of *C. luteus* was constructed using both BI and ML analyses. The mitogenomic sequences of 47 symphytan species were used to construct the phylogeny of *C. luteus* (Cheng et al. 2020; Chen et al. 2020). One consensus tree was obtained, with *C. luteus* identified as one of the basal lineages of family Cimbicidae. The nodes of Bayesian inference (BI) phylogeny tree are provided in Figure 1.

**Figure 1.**
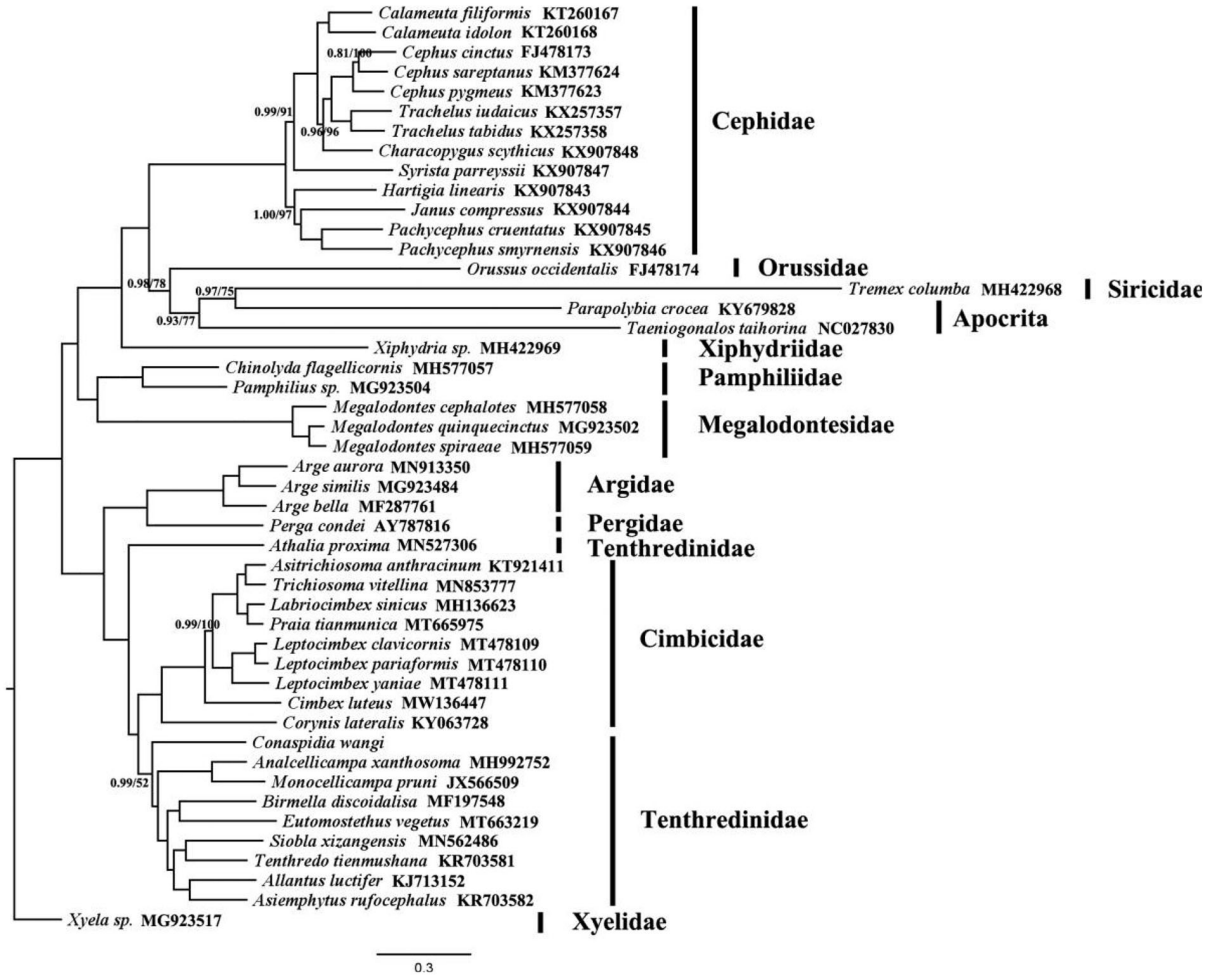
BI and ML approaches were used to construct the phylogenetic tree, based on 13 PCGs. The number on each node is posterior probability. Support values lower than 1.0 in the BI analysis and 100% in the ML analysis were shown. GenBank accession number for each species was provided with species name.

## Data Availability

The data that support the findings of this study are openly available in figshare at https://figshare.com/s/e60e95a596a63f43e6ba.
